# Case Report: Phenotype-Gene Correlation in a Case of Novel Tandem 4q Microduplication With Short Stature, Speech Delay and Microcephaly

**DOI:** 10.3389/fendo.2021.783235

**Published:** 2022-02-03

**Authors:** Umm-Kulthum Ismail Umlai, Basma Haris, Khalid Hussain, Puthen Veettil Jithesh

**Affiliations:** ^1^College of Health and Life Sciences, Hamad Bin Khalifa University, Doha, Qatar; ^2^Department of Pediatric Endocrinology, Sidra Medicine, Doha, Qatar; ^3^Department of Paediatrics, Weill Cornell Medicine - Qatar, Doha, Qatar; ^4^Department of Genetics and Genomics, University College London, London, United Kingdom

**Keywords:** chromosomal duplication, chromosome 4, balanced translocation, rare diseases, short stature, horseshoe kidneys, speech delay, microcephaly

## Abstract

We describe a sporadic case of a pure, tandem, interstitial chromosome 4q duplication, arr[hg19] 4q28.1q32.3 (127,008,069-165,250,477) x3 in a boy born at 36 weeks of gestation. He presented with microcephaly (head circumference <1^st^ percentile), short stature (height <2^nd^ percentile) and poor weight gain (weight <3^rd^ percentile). Hypospadias and horseshoe shaped kidneys were also revealed following a urinary tract ultrasound. Biochemical analysis revealed normal growth hormone and thyroid hormone levels. While gross and fine motor skill development was in line with his age, speech delay was observed. This patient adds to a group of more than 30 cases of pure 4q tandem duplication with common and differing phenotypic presentations. Using a retrospective analysis of previous case studies alongside the current case and bioinformatics analysis of the duplicated region, we deduced the most likely dosage sensitive genes for some of the major phenotypes in the patient. The positive predictive value (PPV) was calculated for each gene and phenotype and was derived by comparing the previously reported patients who have gene duplications and an associated phenotype versus those who had the gene duplications but were unaffected. Thus, the growth retardation phenotype may be associated with *NAA15* duplication, speech delay with *GRIA2* and microcephaly with *PLK4* duplication. Functional studies will help in confirming the observations and elucidating the mechanisms. However, our study highlights the importance of analysing case reports with pure duplications in defining phenotype-gene relationships and in improving our knowledge of the function of precise chromosomal regions.

## 1 Introduction

Chromosome 4q duplication has been reported in over 60 cases (1), most as a result of chromosomal rearrangements which are inherited as unbalanced translocations from unaffected parents (2). Around half of these cases have been reported with a pure, tandem interstitial duplication, i.e., without other chromosomal anomalies, occurring directly after the original arrangement.

Phenotypic variation exists based on the exact location, size and expressivity of the duplicated genes (3). The reported phenotypic features include developmental delay (1, 4, 5), speech and language delay (6), psychomotor retardation (1, 7, 8), epilepsy or seizures (9) intellectual disability (1, 6, 10), microcephaly (3, 10, 11), vision problems (12, 13), growth retardation (4, 14), abnormalities to the extremities (14), retromicrognathia (3, 14, 15), deformed nasal bridge (16, 17), malformed ears (7, 14, 17) and other facial dysmorphisms (1, 11, 16, 18). Larger duplications are sometimes associated with renal or cardiac abnormalities (7, 18, 19). However, elucidating the causes of various phenotypes due to duplication in specific genes in the chromosomal region has been difficult due to the large number of genes affected and the phenotypic diversity.

We analysed a patient with a *de novo* ~38Mb (38,242,408bps) pure, direct interstitial 4q duplication. Pure duplications of this kind are important in establishing phenotype-gene correlations (20). Other types of 4q duplications involving translocations with other chromosomes are not reliable cases to establish such correlations as the new location could influence the resulting phenotype (21). Similarly, duplications involving interstitial inversions within 4q could also cause phenotypic changes, and hence unreliable for causal inference studies (22).

Considering the microduplication and the phenotypic characteristics in our patient, we attempted to elucidate the role those duplicated genes play in growth and development. We hypothesised that the phenotypes which the patient presented could be caused by duplications of the limited number of genes in the 4q region. Using a deductive reasoning approach through the retrospective analysis of previous case studies alongside the current case of pure partial 4q microduplication, we present the most likely casual genes which could be the critical dose sensitive genes in the 4q duplication syndrome.

## 2 Case Presentation

**Figure 1:**
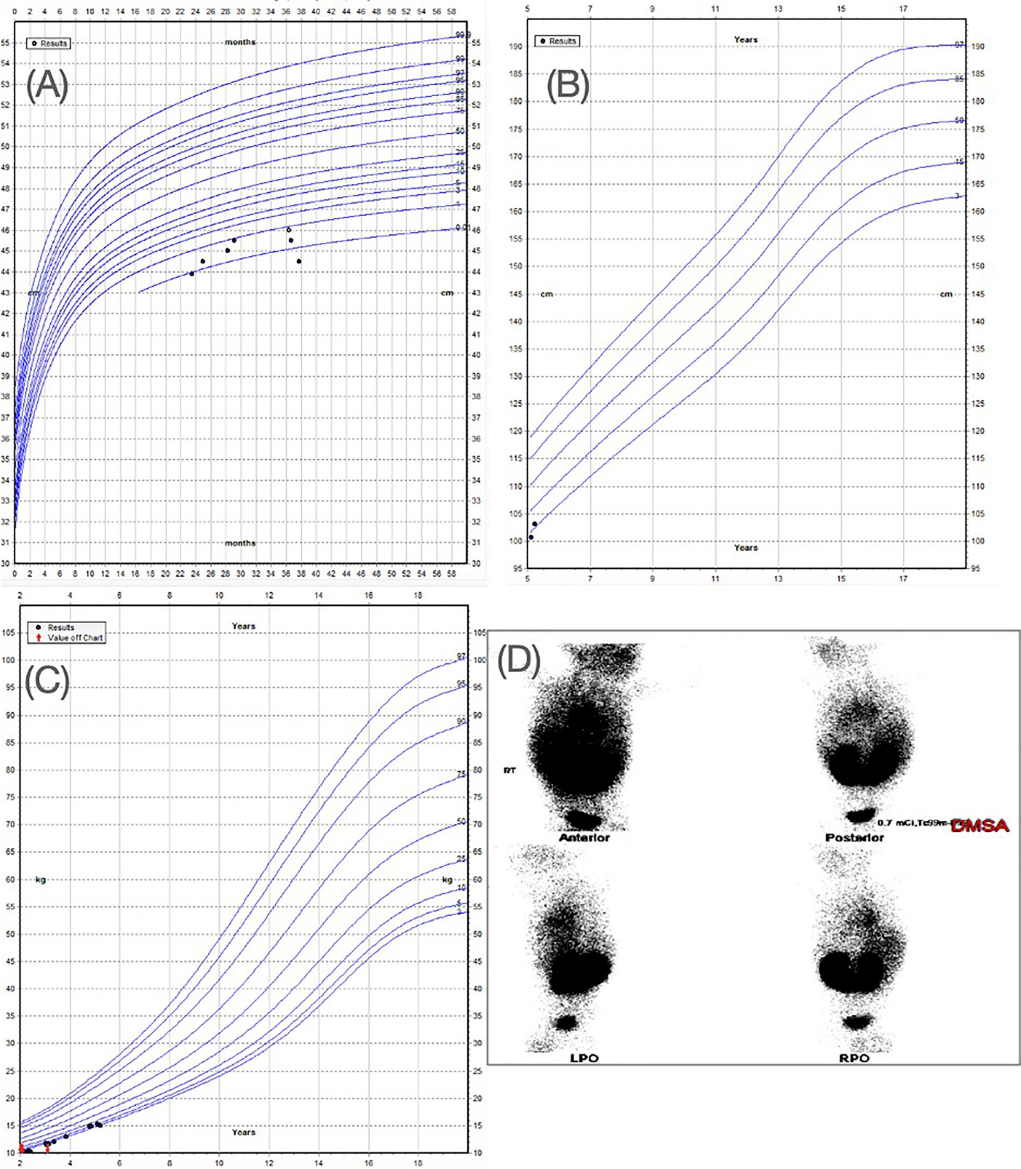
**(A)** WHO head circumference for boys aged 0-5 years. Head circumference of the patient is indicating a value below 1st percentile. **(B)** WHO height-for boys aged 5-19 years showing height of the patient below 2nd percentile. **(C)** WHO Weight for boys aged 2-20 years showing the weight of the patient below the 3rd percentile. **(D)** Renal DMSA showing horse-shoe kidneys (this panel was previously used in a conference poster for the patient case (42).

A male patient presented as a premature child of 36 weeks at 2kg, delivered *via* c-section and admitted to the neonatal intensive care unit for oxygen supply. He had an uneventful gestational period and delivered to a healthy, non-consanguineous couple in their 20s (mother, gravida 3, para 3) and 30s (father), with no relevant medical history. The proband also has two healthy, unaffected siblings. The patient presented with microcephaly demonstrating a head circumference of below the 0.22^nd^ percentile (-3.57SDs). His height was below 1.45^th^ percentile (-2.19SDs) and his weight was at the 2.80^th^ percentile (-1.96 SDs) (**Figures 1A–C**). He exhibited some mild facial dysmorphism with a small face and receding chin.

At the last clinical visit, at the age of 5 years, the patient showed failure to thrive, aggressive behavioural issues and severe speech and language delay. His language capabilities were limited to three-word sentences incomprehensible to strangers. He did demonstrate comprehension for simple, familiar commands from his parents. He did not have any breathing difficulties; heart problems or other medical problem and his gross motor skills were within normal range.

### 2.1 Investigations

Observation of severe chordee with hypospadias and urinary tract infection prompted ultrasound investigations, which revealed the presence of horseshoe kidneys. This was confirmed by a DMSA scan (**Figure 1D**). CT evaluation did not reveal any remarkable findings. The patient did not have any foul-smelling urine or hematuria. He was reported to have a good urine output with a strong urinary stream. The patient’s biochemical analysis revealed normal growth hormone and thyroid hormone levels (**Table 1**).

**Table 1:** Biochemical measurements.

Investigation	Value	Reference range
IGF	104ng/mL	27.4-113.5ng/mL
IGF binding protein 3	3.24 mcg/mL	0.8-3.9 mcg/mL
Sodium	134mmol/L	130 - 150mmol/L
Potassium	4.2mEq/L	3.4 – 4.7mEq/L
BUN	6.0mg/dL	5.0 – 18. 0 mg/dL
Creatinine	29mcmol/L	17- 36mcmol/L
Adjusted Ca	2.41mmol/L	2.2 -2. 7 mmol/L
Chloride	106mEq/L	90 – 110 mEq/L
Random Glucose	4.6mmol/L	4.1- 5.9 mmol/L
Albumin	37g/L	34 – 54 g/L
Growth Hormone	10.40ng/mL	10-50ng/mL
Thyroid (Free T4) TSH	12.3pmol/ L1.35mIU/L	9.5-17.8 0.68- 3.35

### 2.2 Treatment

The patient has not undergone any speech or behavioural therapy, nor has he taken any medication. However, he has undergone initial surgical correction of his hypospadias.

### 2.3 Outcome and Follow-up

The patient’s growth, although initially stunted, has continued to follow a healthy trajectory suitable for his age, ethnicity, and gender. He has been able to catch up in growth without the use of growth hormone treatment. The patient is not currently taking any medications.

The patient’s hypospadias repair was not successful within the first surgery and required two unanticipated further follow up surgeries to correct the deformity. The patient is currently awaiting his third surgery. Cardiac, auditory, and renal surveillance need to be carried out to ensure no additional complications develop as the patient ages. Although rare, the horseshoe kidneys put the child at an increased risk for urinary tract infections, kidney stones and renal tumors, so the child must be monitored for these possible complications.

### 2.4 Genetic Analysis

The patient’s peripheral blood samples were used for microarray analysis, which was performed using the Affymetrix ^®^ CytoScan^®^ 750K platform using 550,000 independent probes (non-polymorphic) as well as 200,000 single nucleotide polymorphisms against the GRCh37 reference genome. Results were validated using high resolution chromosomal G-banding analysis using 11 metaphase cells. Analysis was carried out using Affymetrix^®^ Chromosome Analysis Suite v3.2 software, capable of detecting unbalanced rearrangements greater than 500Kb. Chromosomal microarray analysis revealed a *de novo* duplication in the long arm of chromosome 4 (chr4). The duplication spans the cytogenetic band regions of q28.1 and q32.3 for a total of 38Mb (chr4:127,008,069-165,250,477 GRCh37, 20% of chr4) (**Supplementary Figure S1**).

Genes belonging to the duplication region (4q28.1-32.3) were extracted from the University of California Santa Cruz (UCSC) genome browser (https://genome.ucsc.edu/index.html) using the GRCh37 reference genome coordinates, 44:127,008,069-165,250,477. The Online Mendelian Inheritance in Man (OMIM, https://www.omim.org/) genes with a disorder of known molecular pathology or with duplication/deletion syndromes were selected. Further, genes were filtered based on their association to our patient’s phenotypes (growth delay, microcephaly, renal anomalies, urogenital abnormalities, speech/language delay/impairment). The region had a total of 177 known Ensembl genes, including 153 known coding genes (19% of the coding genes in chr4). Eighty-nine of these genes were present in the OMIM database with an associated genetic disorder, of which 26 had known molecular mechanisms of pathogenicity including deletion/duplication syndromes (**Supplementary Table S1**). Out of the 26 OMIM genes, five genes matched most of the phenotypes of our patient (**Table 2**). Six of the OMIM genes (*GLRB*, *ETFDH*, *LRBA*, *MMAA*, *MFSD8*, *LRAT*) present in the duplication region are known to be dosage sensitive genes associated with a haploinsufficiency disorder, however, some of these were not associated with the patient’s phenotypes, and nothing is known regarding the exact effect of their duplication (triplosensitivity).

**Table 2:** Data showing the candidate genes within the patient’s duplication region 4q 28.1-32.3 with the relevant associated HPO phenotype and OMIM disorder.

Gene Symbol	Full Gene Name	Chr4 Gene Region (GRCh37)	HPO Associations	OMIM Disorder	MT	MT+MN	PPV
*INTU*	Inturned Planar Cell Polarity Protein	128.55-128.64Mb; 4q 28.1	Speech delay	Orofaciodigital syndrome XVII and Short-rib thoracic dysplasia 20 with polydactyly	4	7	0.57
*NAA15*	N-Alpha-Acetyltransferase 15, NatA Auxiliary Subunit	140.22- 140.312; 4q31.1	Speech delay	Intellectual disability, autosomal dominant 50 (poor weight gain, behavioral issues, developmental delay)	6	9	0.67
***GRIA2***	**Glutamate Ionotropic Receptor AMPA Type Subunit 2**	**157.2 – 157.37 Mb; 4q32.1**	**Speech delay**	**Neurodevelopmental disorder with language impairment and behavioral abnormalities**	**9**	**10**	**0.90**
***PLK4***	**Polo Like Kinase 4**	**128.80-128.82Mb; 4q28.1**	**Microcephaly**	**Microcephaly and chorioretinopathy, autosomal recessive, 2**	**5**	**10**	**0.50**
*INTU*	Inturned Planar Cell Polarity Protein	128.55-128.64Mb; 4q 28.1	Short stature	Orofaciodigital syndrome XVII and Short-rib thoracic dysplasia 20 with polydactyly	4	7	0.57
*PLK4*	Polo Like Kinase 4	128.80-128.82Mb; 4q28.1	Short stature	Microcephaly and chorioretinopathy, autosomal recessive, 2	7	10	0.70
***NAA15***	N-Alpha-Acetyltransferase 15, NatA Auxiliary Subunit	**140.22- 140.312; 4q31.1**	**Short stature**	**Intellectual disability, autosomal dominant 50 (poor weight gain, behavioral issues, developmental delay)**	**8**	**10**	**0.80**

HPO, Human Phenotype Ontology; OMIM, Online Mendelian Inheritance in Man database. MT, patients who have a duplication in the gene and exhibit the mentioned phenotype. MN, patients who have a duplication in that gene but do not manifest the phenotype. PPV represents the probability that the duplication of the gene is predictive of the exhibited phenotype [PPV= MT/(MT+MN)]. Some genes are mentioned twice to represent two different phenotypes. Genes in bold are those selected as candidates for correlation to the mentioned phenotype.

#### 2.4.1 PPV Analysis

**Figure 2:**
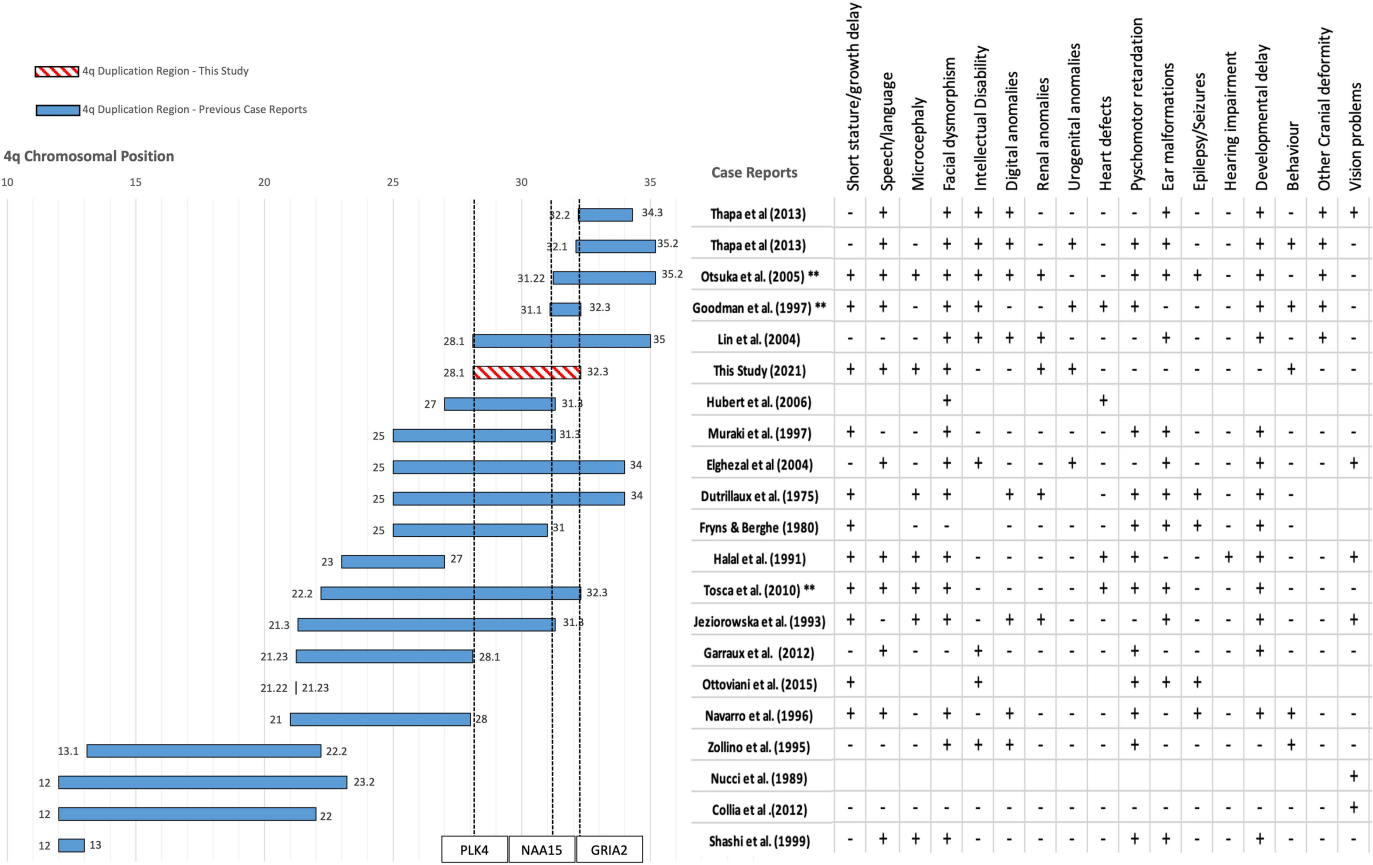
Mapping the duplication region of our patient against 23 other patients of similar pure 4q chromosome duplications from 19 published case reports obtained from literature. Filled bars show the interstitial duplication sizes of previously reported cases. Location is reported as chromosomal band size on the x axis. The axis leading to 0 represents the centromere and the opposite end represents the telomere. Chromosomal coordinates in base pairs (bps) are given in the **Supplementary Table S2**. The two patients from Thapa et al. (2013) each have a distinct duplication. The number of patients affected with each duplication is represented by the number of asterisks * symbols next to the citation on the y axis. The region reported by our study is shown with a pattern filled bar and includes the region of 4q28.1-32.3. The start and end of each region is mentioned as a data label at the beginning and end of each bar. Each case mentioned presents a different combination or spectrum of phenotypes. The table on the right of the plot represents the clinical presentation of specific phenotypes indicated on the first row in each of the case reports. + sign indicates the presence of the phenotype and a – sign indicates absence, blank boxes represent no available information.

We then performed a comparative analysis with all the previous cases reported with 4q duplications to deduce the genes potentially associated with the specific phenotypes seen in the patient (**Figure 2**). All previous case reports of pure interstitial 4q duplications were compared to the current case to obtain a positive predictive value (PPV) of each phenotype-gene correlation:


$$Positive\;Predictive\;Value\;(PPV)=\frac{MT}{MT+MN}$$

where MT is the number of patients with the mutation and presenting the phenotype, and MN is the number of patients with the mutation, but not presenting the phenotype (23). Genes with the highest PPVs for each phenotype were selected as being the most likely critical candidates for the phenotype seen in our patient.

##### 2.4.1.1 Short Stature Phenotype

Out of the five genes within the duplication region which appeared in our gene panel for short stature, *LRBA* does not have an OMIM-phenotype supporting the association with short stature. “Smith-McCort dysplasia” associated with *RAB33B* is more relevant to skeletal dysplasia rather than growth retardation, which our patient presents, and hence these two genes were excluded. Three remaining genes within the duplication region appeared in our gene panel for short stature: *INTU*, *NAA15* and *PLK4*. Mutations in *INTU* are pathogenic in “Orofaciodigital syndrome” and “Short-rib thoracic dysplasia”, autosomal recessive conditions affecting growth; one of which is a skeletal dysplasia- not consistent with our patient’s phenotype. *PLK4* is implicated in autosomal recessive disorder, “Microcephaly and chorioretinopathy 2”, for which varying degrees of dwarfism is a major symptom, while *NAA15* is implicated in the short stature disorder, “Intellectual disability, autosomal dominant 50”. Duplication of *NAA15* produced the highest PPV for the short stature phenotype (0.80) among these genes (**Table 2**).

##### 2.4.1.2 Speech Delay Phenotype

Out of the four genes within the duplication region which appeared in our gene panel for speech delay, the OMIM disease associated with *MFSD8* was not relevant to the speech delay phenotype and so it was excluded. Disorders represented by *INTU* and *NAA15* exhibited varying degrees of speech delay/impairment. While three genes within the duplication region remained in our gene panel for speech delay: *INTU*, *NAA15*, *GRIA2*, the highest PPV was for *GRIA2* (0.90).

##### 2.4.1.3 Microcephaly Phenotype

*PLK4* was the only gene with an associated OMIM microcephaly phenotype within the region of 4q28.1-4q32.1 and had a PPV of 0.50. This indicates it is the most likely cause of microcephaly in at least 50% of the 4q duplication syndrome patients in addition to our patient.

##### 2.4.1.4 Genitourinary and Renal Abnormality Phenotype

We were unable to identify a possible candidate gene responsible for the genitourinary or renal malformations observed in the child.

## 3 Discussion

To our knowledge, this is the first report of potential association of specific genes within the critical region of 4q28.1-32.1 with microcephaly, short stature and speech delay in 4q duplication syndrome. *NAA15*, the gene we identified as potentially associated with short stature from our phenotype-gene correlation analysis, is reported as a dosage sensitive gene on ClinGen, with reports of haploinsufficiency causing disease (24), but there are no reports on gene duplication. Heterozygous missense mutations in *NAA15* were associated with speech, language delay, behavioural abnormality, intellectual disability, short stature, and poor growth (24, 25). *NAA15* is a gene coding for an N-alpha-acetyltransferase 15 which forms a dimeric protein. This protein binds with *NAA10* subunit to make a highly evolutionarily conserved Nat-A complex which is responsible for around 40% of post-translational acetylation modifications of human proteins. Extensive functional studies are lacking for the *NAA1*5/Nat-A complex in humans so it is not certain how *NAA15* could influence short stature or growth retardation. However, the Nat-A complex has been found to be crucial in embryogenesis and cellularization of endosperms in *Arabidopsis* (26–28) as well as in the early development of *Danio Rario* (29). Other studies have linked *NAA10* with several cellular signalling pathways, including the JAK-STAT (30) and MAPK (31) pathways, which are known to be involved in GH-IGF-1 growth cascades. This suggests that *NAA15* and the Nat-A complex are crucial in normal cell functioning (32) and that loss of function of this gene may decrease post-translational modifications on proteins involved in cellular processes leading to abnormal development.

Furthermore, it is not clear how overabundance of *NAA15* could lead to pathogenesis. However according to one theory on the pathogenesis of CNVs affecting protein complex subunits; the overabundance of a single subunit from a complex may lead to a stoichiometric ratio imbalance leading to aberrant complex formation and or nonsense mediated decay (33–35). This could explain why we see a similar phenotype with both the loss and duplication of *NAA15*. The best way to establish this hypothesis would be to recapitulate the duplication in an *in vitro* cell line using patient-derived fibroblast cells or to recreate human pluripotent cells from the patient’s blood and to measure the level and size of the Nat-A complex being expressed compared to wild type controls.

PPV analysis supported *GRIA2* as correlated with the speech delay phenotype in our patient. The gene itself lies 7.79Mbps away from the patient’s duplication breakpoint so it is unlikely to be disrupting the gene function. Mutations affecting *GRIA2* are specifically responsible for causing neurodevelopmental disorders with language and behavioral abnormalities. Copy number variations of *GRIA2* are generally rare, however it has been proposed that haploinsufficiency (deletions) of this gene may be pathogenic (36, 37). *GRIA2* functions as a subunit for a glutamate cation channel receptor GluR2 in the brain and is activated by AMPA neurotransmitter binding; it plays a role in several neurological processes. Overexpression of GluR2 receptors could mean modification in Ca^2+^ permeability and conductivity of the ions (38). Mutations in the *GRIA4*, another receptor from the same GluR family, affect the gaining of long-term spatial reference memory (38). Similarly, duplication of *GRIA2* could affect the dosage amount and levels of receptors available, negatively impacting the ability of individuals to learn and store language memory leading to speech delay.

*PLK4* encodes for Polo Like Kinase 4, a protein that regulates centriole amplification during the cell division. The gene lies 0.872Mbps away from the patient’s duplication endpoint so it is unlikely to be disrupting the gene function. Pathogenic, homozygous frameshift and missense mutations in *PLK4* often damage centriole biogenesis, microtubule spindle formation during mitosis, cell proliferation, viability and cause abnormal cytoskeleton formation (39–41). Specifically, these mutations often lead to increased cell mortality due to inefficient mitosis. Since *PLK4* is pathogenic in a loss of function, autosomal recessive fashion, it is difficult to interpret how centriole biogenesis and mitosis could be compromised if the protein is duplicated and overexpressed.

Although renal and urinary system abnormalities were reported in previous cases, horseshoe kidneys seen in our patient were not shared by these probands. This suggests duplications of genes between 4q28.1-31.1 to be potentially associated with this phenotype.

The small number of patients with a duplication in this region is a limitation of the study; having a larger group of patients to compare to would strengthen the correlations derived from the analysis. Copy Number Variant (CNV) cases such as the one from this study are often recorded in the DECIPHER database. For this 4q region we found multiple patient cases with pure duplications in the database. However, the clinical information for the patients were insufficient, so they were not included in the present study. In the case of a similar study, the authors would contact the submitters for each of these cases and to obtain complete information for each patient. In addition, the possible effect of epistasis within this duplication region cannot be excluded at present, which may interfere with the attribution of a single gene per phenotype. Especially since some of the patients do not share the same phenotypes even though they share the same duplication region. Although this could also be due to incomplete penetrance or variable expressivity.

Further work is required to understand the exact mechanisms at play resulting from the triplosensitivity, since several mechanisms are known to responsible for leading to microcephaly, short stature and speech delay. In addition, the role of some the genes included in the duplication region remain to be elucidated; these could also be playing a role in the pathogenicity, but we cannot know unless further investigation is performed for those other genes. To validate our estimations on gene dosage pathogenicity, further experiments in cell and animal models to assess for phenotypic abnormalities are required. However, our study highlights the importance of analysing case reports with pure duplications in defining phenotype-gene relationships and in improving our knowledge of the function of precise chromosomal regions.

## 4 Patient Perspective

The patient’s father expressed his satisfaction at the current course of medical consultation. He was pleased that his son’s behavior with others and progress at school was improving. He noted how his son has become more social and has become clearer at speech. He did express his concern regarding his son’s surgical correction of hypospadias and noted that he was not happy that it was taking multiple surgeries for the repair to be completed. The patient’s father hopes that one day there will be treatment for genetic disorders such as his son’s.

## Data Availability Statement

The original contributions presented in the study are included in the article/**Supplementary Material**. Further inquiries can be directed to the corresponding author.

## Ethics Statement

The studies involving human participants were reviewed and approved by Sidra Medicine. Written informed consent to participate in this study was provided by the participants’ legal guardian/next of kin. Written informed consent was obtained from the patient’s legal guardian/next of kin for the publication of any potentially identifiable images or data included in this article.

## Author Contributions

U-KU consented the family for publication, collected and analyzed the data and wrote the manuscript. KH supervised the clinical aspects of the study and contributed to writing. BH recruited the patient, collected blood samples and supported in writing. PJ supervised the work and wrote the manuscript. All the authors read and approved the final manuscript.

## Funding

U-KU received PhD scholarship and PJ received research funds from the College of Health & Life Sciences, Hamad Bin Khalifa University, Qatar.

## Conflict of Interest

The authors declare that the research was conducted in the absence of any commercial or financial relationships that could be construed as a potential conflict of interest.

## Publisher’s Note

All claims expressed in this article are solely those of the authors and do not necessarily represent those of their affiliated organizations, or those of the publisher, the editors and the reviewers. Any product that may be evaluated in this article, or claim that may be made by its manufacturer, is not guaranteed or endorsed by the publisher.
