# Supplementary material for: Case Report: Phenotype-Gene Correlation in a Case of Novel Tandem 4q Microduplication With Short Stature, Speech Delay and Microcephaly
Source: Front Endocrinol (Lausanne). 2022 Feb 3;12:783235. doi: 10.3389/fendo.2021.783235 (PMC8851600; doi:10.3389/fendo.2021.783235)
Supplement: Supplementary file 1 [file DataSheet_1.docx]

Supplementary Material

# Supplementary methods

## Duplication region gene compilation:

The following coordinates 4:127,008,069-165,250,477 were input into the "Position" box on the GRCh37/hg19 University of California Santa Cruz (UCSC) genome browser [1]. Genes that are located within this duplicated region of 4q 28.1-32.3 were shortlisted and filtered according to genes with OMIM entries. The table tool browser from UCSC browser function was utilized to filter only the OMIM genes with a disorder of known molecular pathology or with duplication/deletion syndromes. The chromosome start and end sites, name, OMIM gene name and description as well as inheritance mode fields were selected; "chromStart", "chromEnd", "name", "approvedGeneSymbol", "omimID", "description", "inhMode". The final table was download and the entries were compared to the curated phenotype gene panels.

## Gene panel curation:

Genes associated with growth delay were compiled based on keyword searches ("growth hormone deficiency", "isolated growth hormone deficiency", "growth hormone", "short stature", "hypopituitarism", "delayed bone age", "IGHD", "GHRHR", "GHR", "GH1") from online databases from the Human Gene Mutation Database (HGMD)[2] and Online Mendelian Inheritance of Man [3] and National Centre for Biotechnology Information [4]between October 2018- December 2018. The list of genes occurring in the duplicated region were compared to each gene panel to return possible candidates for each phenotype. To obtain a list of genes associated with kidney abnormalities and horseshoe kidney phenotype, a search was conducted on Human Phenotype Ontology (HPO) using search terms "horseshoe kidneys" HPO:000085 and "abnormality of the kidney" HP:0000077 respectively (on Sunday 20^th^ June 2021). A clinical genetics gene panel obtained from Blueprint Genetics [5] was also used for “renal malformations". A similar search was conducted on HPO to obtain a list of "speech delay" (HP:0000750) disease genes (Sunday 20^th^ June 2021).

## Candidate Dosage-Sensitive Genes and Critical Region determination

Genes obtained from Step (1) Duplication region gene compilation were compared to the gene panel curated from Step (2) to determine whether any of the duplicated genes have any associations to the relevant phenotypes.

### Gene enrichment

To assess the gene enrichment of the list duplicated genes in our patient, we input all 177 duplicated genes into Enrichr.com [6] a gene set enrichment analysis tool and identified the HPO terms, GO biological and molecular function pathways [7][8], Orphanet diseases [9] and KEGG pathways [10] enriched by these genes. The top 10 enriched terms/ phenotypes were selected and ordered by P-values using the bar graph and table feature.

### Gene-Phenotype Relationship

The following search terms were also input into Phenolyzer [11]: 1) behavioral developmental 2) growth delay 3) horseshoe kidney 4) horseshoe kidneys 5) hypospadias 6) microcephaly 7) psychiatric abnormality 8) short stature 9) developmental delay and congenital heart defects 10) short stature 11) short syndrome 12) speech delay in order to generate a pictogram which helps to visualize the gene associations of these phenotypes.

A similar search was conducted with Phenomizer [12], an online genotype-phenotype matching tool with the following search terms: "Failure to thrive (HP:0001508)", "Horseshoe kidney (HP:0000085)", "Severe short stature (HP:0003510)", "Microcephaly (HP:0000252)", "Delayed speech and language development (HP:0000750)", "Hypospadias (HP:0000047)" and "Global developmental delay (HP:0001263)"; with input settings of sporadic inheritance.

### Literature collection

All published cases of distal 4q duplication were collected using PubMed between 29^th^ June-7^th^ September 2021 and search terms “4q duplication”, “distal 4q duplication”, “4q trisomy duplication”, “Chromosome 4 duplication syndrome”, “chr4q duplication”, "4q trisomy", “4q interstitial duplication” and filtered by case reports, review papers and journal articles. Search results were manually filtered by cases representing only pure, direct duplications within chromosome 4q and without any other chromosomal abnormalities, including inversions or translocations at other chromosomes. Literature searches were limited to availability of English translation. Cases without clear exclusions of other abnormalities and those with unclear band breakpoints were disregarded. Phenotypes of all patients plus exact known coordinates or bands of anomalies were collected and tabulated using Microsoft Excel.

### Positive predictive value calculation

A score of 0 or 1 was given for each of the reported phenotypes for each of the candidate genes matching the phenotype gene lists. Each phenotype was scored at the end for a total. The number of patients exhibiting each phenotype at a gene locus was divided by the total number of patients with a duplication at that gene locus to give the PPV: Positive predictive value. A separate score was given for each reported phenotype for each gene. Genes that were associated with more than one phenotype had separate scores calculated. Finally, the genes with the highest scores were accepted as the most likely contributors to the phenotype in the affected patients.

# Supplementary Results:

## Gene enrichment:

The top 10 results from the gene enrichment analysis did not yield any supporting evidence for the phenotypes presented in our patient. None of the pathways or diseases were indicative or consistent with the clinical case.

## Gene-Phenotype Relationship:

Phenolyzer results did not produce any meaningful or reproducible associations between duplicated genes and associated phenotypes. Phenomizer disease results also did not bring up genes present within the duplication region as related to the patient's phenotypes. The first three disease results were chromosomal deletion syndromes; Chromosome 3q29 deletion syndrome (OMIM# 609425), Chromosome 15q26-qter deletion (OMIM# 612626, culprit gene is MCTP2) syndrome and chromosome 10q26 deletion syndrome (OMIM #219150) with p-values of 0.0432, 0.00834 and 0.1154 respectively, which are not consistent with the patient's genetic findings. The 4^th^ finding describes a disorder named Cutis Laxa, Corneal Clouding and Mental retardation (OMIM: 219150). Further disease results brought up by the tool were other chromosomal abnormalities; Chromosome 22q11.2 duplication syndrome (OMIM# 608363), Chromosome 17p11.2 duplication syndrome (OMIM# 610883).

## Literature collection:

Case report results were obtained using the aforementioned search terms. After filtration, the number of relevant cases reached 19 (total of 23 patients). The cases were limited to only those of pure/direct, interstitial chromosome 4q duplication. Cases with any other chromosomal anomalies were discarded, including inversions or unbalanced translocations involving other chromosomes.

# Supplementary References:

1 Kent WJ, Sugnet CW, Furey TS, Roskin KM, Pringle TH, Zahler AM, Haussler a. D. The Human Genome Browser at UCSC. *Genome Res* 2002;**12**:996–1006.

2 Stenson PD, Ball E V., Mort M, Phillips AD, Shiel JA, Thomas NST, Abeysinghe S, Krawczak M, Cooper DN. Human Gene Mutation Database (HGMD ^®^ ): 2003 update. *Hum Mutat* 2003;**21**:577–81.

3 Johns Hopkins University. OMIM - Online Mendelian Inheritance in Man. 2020.https://omim.org/ (accessed 28 Jun2020).

4 NCBI Resource Coordinators*. Database resources of the National Center for Biotechnology Information . *Nucleic Acids Res* 2015;**43**:D6–17.

5 Blueprint Genetics. Renal Malformation Panel. https://blueprintgenetics.com/ (accessed 15 Nov2021).

6 Chen EY, Tan CM, Kou Y, Duan Q, Wang Z, Meirelles G V., Clark NR, Ma’ayan A. Enrichr: Interactive and collaborative HTML5 gene list enrichment analysis tool. *BMC Bioinformatics* 2013;**14**:128.

7 Ashburner et al. Gene ontology: tool for the unification of biology. Nat Genet. May 2000;25(1):25-9.

8 The Gene Ontology resource: enriching a GOld mine. Nucleic Acids Res. Jan 2021;49(D1):D325-D334.

9 Orphanet: an online database of rare diseases and orphan drugs. Copyright, INSERM 1997. Available at http://www.orpha.net Accessed 15 November 2021.

10 Kanehisa M, Goto S. KEGG: Kyoto Encyclopedia of Genes and Genomes. 2000. http://www.genome.ad.jp/kegg/ (accessed 10 May2020).

11 Yang H, Robinson PN, Wang K. Phenolyzer: phenotype-based prioritization of candidate genes for human diseases HHS Public Access. *Nat Methods* 2015;**12**:841–3.

12 Köhler S, Bauer S, Horn D, Robinson PN. Walking the Interactome for Prioritization of Candidate Disease Genes. *Am J Hum Genet* 2008;**82**:949–58.

# Supplementary Figures and Tables

## Supplementary Figures

**Supplementary Figure 1.** Figure depicting the duplication region of the bands q28.1-q32.3 on chromosome 4. Interstitial duplication of size **38,242,408 bps** with 177 Ensembl genes duplication within this region.


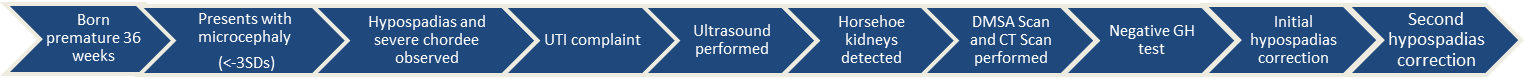


**Case study enrollment**

Supplementary Figure 2: Chronology of care for the 4q duplication patient from birth to case study enrollment

## Supplementary Tables

| **Supplementary Table S1:**  **Table showing a list of 26 genes with OMIM disorder entries within the region of Chr 4:** 127,008,069-165,250,477 (4q28.1-32.3). Obtained using UCSC genome browser version GRCh37/hg19 (Feb 2009). The total number of 177 UCSC genes of which are 89 OMIM genes duplicated in this region, however only 26 of these are disorders for which there is a known molecular basis, including deletion/duplication syndromes. | | | | | | | |
| --- | --- | --- | --- | --- | --- | --- | --- |
|  | **Gene symbol** | **Full Gene Name** | **Transcription start site (bp)** | **Transcription Stop Site(bp)** | **OMIM ID** | **OMIM disorder** | **Inheritance pattern** |
| 1 | INTU | Inturned Planar Cell Polarity Protein | 128554111 | 128647892 | 610621 | Short-rib thoracic dysplasia 20 with polydactyly/ Orofaciodigital syndrome XVII | AR |
| 2 | PLK4 | Polo Like Kinase 4 | 128802047 | 128820377 | 605031 | Microcephaly and chorioretinopathy | AR |
| 3 | MFSD8 | Major Facilitator Superfamily Domain Containing 8 | 128838886 | 128887118 | 611124 | Macular dystrophy with central cone involvement/ Ceroid lipofuscinosis neuronal 7 | AR |
| 4 | GAB1 | GRB2 Associated Binding Protein 1 | 144258028 | 144395718 | 604439 | Deafness 26 | AR |
| 5 | MMAA | Metabolism Of Cobalamin Associated A | 146540536 | 146581185 | 607481 | Methylmalonic aciduria vitamin B12-responsive cblA type | AR |
| 6 | SLC10A7 | Solute Carrier Family 10 Member 7 | 147175132 | 147443092 | 611459 | Short stature, amelogenesis imperfecta and skeletal dysplasia with scoliosis | AR |
| 7 | TTC29 | Tetratricopeptide Repeat Domain 29 | 147627789 | 147867038 | 618735 | Spermatogenic failure 42 | AR |
| 8 | EDNRA | Endothelin Receptor Type A | 148402068 | 148466106 | 131243 | Mandibulofacial dysostosis with alopecia/ Resistance to Migraine | AD |
| 9 | NR3C2 | Nuclear Receptor Subfamily 3 Group C Member 2 | 148999914 | 149365850 | 600983 | Pseudohypoaldosteronism type I/ Hypertension (early-onset with exacerbation in pregnancy) | AD |
| 10 | NAA15 | N-Alpha-Acetyltransferase 15, NatA Auxiliary Subunit | 140222658 | 140312538 | 608000 | Mental retardation AD 50 | AD |
| 11 | RAB33B | Member RAS Oncogene Family | 140375274 | 140397763 | 605950 | Smith-McCort dysplasia 2 | AR |
| 12 | LRBA | LPS Responsive Beige-Like Anchor Protein | 151185587 | 151936879 | 606453 | Immunodeficiency 8 with autoimmunity common variable | AR |
| 13 | MAB21L2 | Mab-21 Like 2 | 151503302 | 151505845 | 604357 | Microphthalmia/coloboma and skeletal dysplasia syndrome | AR/ AD |
| 14 | GATB | Glutamyl-TRNA Amidotransferase Subunit B | 152591660 | 152682159 | 603645 | Combined oxidative phosphorylation deficiency 41 | AR |
| 15 | TRIM2 | Tripartite Motif Containing 2 | 154073314 | 154260469 | 614141 | Charcot-Marie-Tooth disease type 2R | AR |
| 16 | TLR2 | Toll Like Receptor 2 | 154605431 | 154627412 | 603028 | Susceptibility to colorectal cancer susceptibility/ Susceptibility to Mycobacterium tuberculosis, Susceptibility to Leprosy | AD |
| 17 | FGB | Fibrinogen Beta Chain | 155484162 | 155493957 | 134830 | Dysfibrinogenemia congenital Afibrinogenemia congenital Hypofibrinogenemia congenital | AR |
| 18 | FGA | Fibrinogen Alpha Chain | 155504277 | 155511894 | 134820 | Dysfibrinogenemia congenital / Amyloidosis familial visceral/ Hypodysfibrinogenemia congenital/ Afibrinogenemia congenital | AD/ AR |
| 19 | FGG | Fibrinogen Gamma Chain | 155525285 | 155533960 | 134850 | Hypofibrinogenemia congenital Hypodysfibrinogenemia Dysfibrinogenemia congenital Afibrinogenemia congenital | AR |
| 20 | LRAT | Lecithin retinol acyltransferase | 155661992 | 155674271 | 604863 | Retinal dystrophy (early-onset) / Severe Leber congenital amaurosis 14 / Retinitis pigmentosa juvenile | AR |
| 21 | GUCY1A1 | Guanylate Cyclase 1 Soluble Subunit Alpha 1 | 156587999 | 156658209 | 139396 | Moyamoya 6 with achalasia | AR |
| 22 | TDO2 | Tryptophan 2,3-Dioxygenase | 156824847 | 156841558 | 191070 | Hypertryptophanemia | AR |
| 23 | GLRB | Glycine receptor subunit beta | 157997276 | 158093242 | 138492 | Hyperekplexia 2 | AR |
| 24 | GRIA2 | Glutamate Ionotropic Receptor AMPA Type Subunit 2 | 158141271 | 158287226 | 138247 | Neurodevelopmental disorder with language impairment and behavioral abnormalities | AD |
| 25 | ETFDH | Electron Transfer Flavoprotein Dehydrogenase | 159593447 | 159630775 | 231675 | Glutaric acidemia IIC | AR |
| 26 | RAPGEF2 | Rap guanine nucleotide exchange factor 2 | 160025202 | 160281325 | 609530 | Epilepsy familial adult myoclonic 7 | AD |
